# Exploring the optimum nitrogen partitioning to predict the acclimation of C_3_ leaf photosynthesis to varying growth conditions

**DOI:** 10.1093/jxb/ery277

**Published:** 2018-07-25

**Authors:** Xinyou Yin, Ad H C M Schapendonk, Paul C Struik

**Affiliations:** 1 Centre for Crop Systems Analysis, Department of Plant Sciences, Wageningen University & Research, AK Wageningen, The Netherlands; 2 Photosyntax B.V., Englaan, EW Wageningen, The Netherlands

**Keywords:** Acclimation, chlorophyll, electron transport, modelling, optimization, photosynthesis, Rubisco

## Abstract

The distribution of leaf nitrogen among photosynthetic proteins (i.e. chlorophyll, the electron transport system, Rubisco, and other soluble proteins) responds to environmental changes. We hypothesize that this response may underlie the biochemical aspect of leaf acclimation to the growth environment, and describe an analytical method to solve optimum nitrogen partitioning for maximized photosynthesis in C_3_ leaves. The method predicts a high investment of nitrogen in Rubisco under conditions leading to excessive energy supply relative to metabolic demand (e.g. low temperature, high light, low nitrogen, or low CO_2_). Conversely, more nitrogen is invested in chlorophyll when the energy supply is limiting. Overall, our optimization results are qualitatively consistent with literature reports. Commonly reported changes in photosynthetic parameters with growth temperature were emergent properties of the optimum nitrogen partitioning. The method was used to simulate dynamic acclimation under varying environmental conditions, using first-order kinetics. Simulated diurnal patterns of leaf photosynthetic rates as a result of acclimation differed greatly from those without acclimation (*A*_without_). However, differences in predicted photosynthesis integrated over a day or over the growing season from *A*_without_ depended on the value of the kinetic time constant (τ), suggesting that τ is a critical parameter determining the overall impact of nitrogen distribution on acclimated photosynthesis.

## Teaser

An analytical solution to the optimum partitioning of leaf nitrogen among photosynthetic proteins is developed, providing a new simple avenue of modelling leaf photosynthetic acclimation to environmental conditions during growth.

## Introduction

One of the major underlying components in predicting ecosystem productivity and crop yield is to model photosynthesis of individual leaves in a canopy under fluctuating environmental conditions. A prevailing approach is to use the steady-state photosynthesis model of Farquhar, von Caemmerer, and Berry (Farquhar *et al.*, 1980; ‘the FvCB model’ hereafter). This model predicts photosynthesis as the minimum of the ribulose-1,5-bisphosphate (RuBP)-saturated rate of CO_2_ assimilation, which is a function of the maximum carboxylation capacity of Rubisco (*V*_c,max_), and the RuBP regeneration-limited rate, which is a function of the maximum electron transport (*J*_max_) (see Supplementary Appendix A at *JXB* online).

A substantial body of experimental work has shown a strong empirical correlation between *V*_c,max_ or *J*_max_ and leaf nitrogen content (e.g. Harley *et al.*, 1992; Walcroft *et al.*, 1997; Kattge *et al.*, 2009). This is expected because nitrogen is a constituent of many functional protein groups of photosynthesis, such as components of the electron transport chain and enzymes of the Calvin cycle (Evans, 1989). Because nitrogen availability is often limiting to plant growth, it is beneficial, in terms of photosynthetic carbon gain, for plants to use nitrogen efficiently. An investment of nitrogen in a protein compound within a leaf ‘appropriate’ to its environment must be of adaptive significance (Walters, 2005).

There are many reports on photosynthetic acclimation to growth environments, such as irradiance and nitrogen availability (Hikosaka and Terashima, 1996; Warren and Adams, 2001), temperature (Yamasaki *et al.*, 2002; Yamori *et al.*, 2005), and CO_2_ levels (Medlyn, 1996; Sharwood *et al.*, 2017). Smith and Dukes (2013) defined acclimation as ‘a physiological, structural, or biochemical adjustment by an individual plant in response to environmental stimulus that is manifested as alternation in the short-term response function of a physiological process’. Evans and Poorter (2001) indicated that to acclimate to their growth environments, plants adjust both biochemical and morphological traits in order to maximize carbon gain. However, in most existing uses of the FvCB model in predicting ecosystem productivity (e.g. Leuning *et al.*, 1995; Lloyd and Farquhar, 1996) and crop growth (e.g. Yin and Struik, 2017; Wu *et al.*, 2018), photosynthetic parameters (e.g. *V*_c,max_ and *J*_max_) are related to overall leaf nitrogen content.

From a meta-analysis of data, Hikosaka *et al.* (2006) and Kattge and Knorr (2007) showed that most parameters of the FvCB model varied with growth temperature. For example, Hikosaka *et al.* (2006) showed that activation energy of *V*_c,max_ (a parameter showing the sensitivity to measurement temperature, see Equation A6 in Supplementary Appendix A) increased 1010 J mol^−1^ per °C increase in growth temperature, explaining a large part of the observed increase of the optimum temperature of photosynthesis with the temperature during growth. Therefore, many researchers stressed the need to incorporate this acclimation response to growth environmental variables into ecosystem models (Smith and Dukes, 2013) and crop models (Yin and Struik, 2010). However, any attempt to incorporate this response has been empirically based on experimental (Bernacchi *et al.*, 2003) or meta-analysis results (Kattge *et al.*, 2009; Friend, 2010; Stinziano *et al.*, 2018). This empirical approach is understandable because acclimation is a complex phenomenon involving all physiological, structural, or biochemical adjustments that probably have different time scales. We hypothesize that the partitioning of nitrogen among photosynthetic components underlies the biochemical aspects of acclimation; to analyse the acclimation response of photosynthetic components to environmental changes, it is necessary first to model leaf photosynthesis on the basis of the nitrogen contents in individual compounds. The analysis of nitrogen costs of photosynthetic compounds and their relationships (e.g. Evans, 1989) made it possible to develop such models.

Several studies (Friend, 1991; Hikosaka and Terashima, 1995; Medlyn, 1996; Hikosaka, 1997) have modelled the optimum nitrogen allocation, on the basis of the nitrogen cost of individual photosynthetic compounds. Friend (1991) separated the photosynthetic nitrogen between two compartments (Rubisco and chlorophyll) only. Medlyn (1996) divided the photosynthetic nitrogen into four pools. While Hikosaka and Terashima (1995, 1996) divided the photosynthetic nitrogen into 5–6 protein complexes (core and light-harvesting complexes of PSI, light-harvesting complex II, core complex of PSII, Rubisco, and electron transport and other Calvin cycle enzymes), their analysis used an empirical hyperbolic equation for the light response curve of leaf photosynthesis. Hikosaka (1997) extended the approach by using the FvCB model. Both Medlyn (1996) and Hikosaka (1997) used a numerical routine to determine the optimum nitrogen partitioning that maximizes daily photosynthesis. These studies highlighted the importance of predicting the nitrogen partitioning to understand photosynthetic acclimation with respect to nitrogen use, but did not model acclimation itself. As acclimation is a process in which photosynthetic compounds adjust from their actual level towards their optimum at a given condition (Kirschbaum *et al.*, 1998), the first step in modelling acclimation is to determine the optimum nitrogen partitioning for a specific environmental condition.

In this study, we first develop a simple method that analytically resolves the optimum nitrogen partitioning among photosynthetic compounds in C_3_ plants. We then examine to what extent the optimum nitrogen partitioning can explain the experimentally observed acclimation of leaf photosynthesis to environmental variables during growth. Finally, we analyse the potential difference between a model considering dynamic acclimation and the prevailing modelling approach that ignores the acclimation of photosynthesis under field environments.

## Materials and methods

### Modifying the FvCB model

Our method for determining the optimum nitrogen partitioning is based on the FvCB model for C_3_ species (Supplementary Appendix A, with all variables listed in Table 1). In order to find the optimum solution to the nitrogen partitioning, we need to simplify the original non-rectangular formula, Equation (A4) in Supplementary Appendix A, into a Blackman-type equation:

**Table 1.** List of variables used in the model

Variable	Definition	Unit
*a*_c_	Coefficient in Equation 2	mmol chlorophyll mol^−1^N
*a*_J_	Coefficient in Equation 3	μmol electron mol^−1^ N s^−1^
*A*	Net leaf photosynthesis rate	μmol CO_2_ m^−2^ s^−1^
*b*_c_	Coefficient in Equation 2	mmol chlorophyll m^−2^
*C*_a_	Atmospheric CO_2_ concentration	μmol mol^−1^
*C*_c_	Chloroplast CO_2_ concentration	μmol mol^−1^
*D*_J_	Energy of deactivation for *J*_max_ in Equation A7	J mol^−1^
*E*	Activation energy for *V*_c,max_, or *K*_mC_, or *K*_mO_	J mol^−1^
*E*_Jmax_	Activation energy for *J*_max_	J mol^−1^
*f*_cyc_	Fraction of cyclic electron transport around PSI	–
*I*_abs_	Absorbed photosynthetically active irradiance	μmol photon m^−2^ s^−1^
*I*_inc_	Incident photosynthetically active irradiance	μmol photon m^−2^ s^−1^
*J*	Rate of linear whole-chain electron transport	μmol electron m^−2^ s^−1^
*J*_max_	Maximum value of *J* under a saturating irradiance	μmol electron m^−2^ s^−1^
*J*_max25_	*J*_max_ at 25 °C	μmol electron m^−2^ s^−1^
*J*_ (*T*)_	Function for the temperature dependence of *J*_max_	–
*K*_C25_	Specific activity of Rubisco at 25 °C	g CO_2_ g^−1^ Rubisco s^−1^
*K*_mC_	Michaelis–Menten constant of Rubisco for CO_2_	μmol mol^−1^
*K*_mC25_	*K*_mC_ at 25 °C	μmol mol^−1^
*K*_mO_	Michaelis–Menten constant of Rubisco for O_2_	mmol mol^−1^
*K*_mO25_	*K*_mO_ at 25 °C	mmol mol^−1^
*k*_S_	Coefficient in Equation 5	mol N s μmol^−1^electron
*N*_C_	Leaf nitrogen allocated to chlorophyll	mol N m^−2^
*N*_leaf_	Total nitrogen content in leaves	mol N m^−2^
*N*_leafE_	Physiologically effective total nitrogen content in leaves	mol N m^−2^
*N*_photo_	Photosynthetic nitrogen content in leaves	mol N m^−2^
*N*_R_	Leaf nitrogen allocated to Rubisco	mol N m^−2^
*N*_S_	Leaf nitrogen allocated to other soluble protein	mol N m^−2^
*N*_T_	Leaf nitrogen allocated to electron transport system	mol N m^−2^
*O*	Oxygen concentration (ambient level=210)	mmol mol^−1^
*R*	Universal gas constant (=8.314)	J K^−1^ mol^−1^
*R*_d_	Day respiration rate	μmol CO_2_ m^−2^ s^−1^
*S*_c/o25_	Relative CO_2_/O_2_ specificity of Rubisco at 25 °C	mmol μmol^−1^
*S*_J_	Entropy term for *J*_max_ in Equation A7	J K^−1^ mol^−1^
*T*	Leaf temperature	°C
*T*_opt_	Optimum leaf temperature for Φ_2LL_	°C
*V*_c_	Rubisco activity-limited carboxylation rate	μmol CO_2_ m^−2^ s^−1^
*V*_c(*C*c)_	Function for the *C*_c_ dependence of *V*_c_	–
*V*_c,max_	Maximum velocity of Rubisco-limited carboxylation	μmol CO_2_ m^−2^ s^−1^
*V*_c,max25_	*V*_c,max_ at 25 °C	μmol CO_2_ m^−2^ s^−1^
*V*_c(*T*)_	Function for the temperature dependence of *V*_c,max_	–
*V*_j_	Electron transport-limited carboxylation rate	μmol CO_2_ m^−2^ s^−1^
*V*_j(*C*c)_	Function for the stoichiometry of electron transport	μmol CO_2_ μmol^−1^electron
α	Efficiency of PSII electron transport on basis of *I*_abs_	mol e^−^ mol^−1^ photon
ε	Quantum efficiency for CO_2_ fixation	mol CO_2_ mol^−1^ photon
Φ_2LL_	Quantum efficiency for PSII electron transport	mol e^−^ mol^−1^ photon
Φ_2LL,max_	Maximum value of that is achieved at *T*_opt_	mol e^−^ mol^−1^ photon
Φ_2LL_/Φ_1LL_	PSII to PSI electron transport efficiency ratio	–
ν	Coefficient to convert *N*_R_ to Rubisco	g Rubisco mol^−1^ N
*τ*	Time constant	min
Γ_*_	CO_2_ compensation point in the absence of day respiration	μmol mol^−1^
Ω	Gap between *T*_opt_ and temperature at which Φ_2LL_=Φ_2LL,max_/*e*	°C

$$J=\text{min}(\text{a}I_{\text{abs}},J_{\text{max}})$$ (1)

where α is defined according to Yin *et al*. (2004). There is experimental support for the linearity between *J* and *I*_abs_ within the moderate range of irradiance (Cheeseman and Lexa, 1996; see the Discussion).

### Nitrogen dependence of component processes

Following Medlyn (1996), we divide total photosynthetic leaf nitrogen (*N*_photo_) into four pools. Two of them are in the thylakoid: nitrogen required for the chlorophyll–protein complex (*N*_C_) and for components of the electron transport system (*N*_T_). The other two are soluble proteins related to the activities of the Calvin cycle enzymes, which are divided between Rubisco (*N*_R_) and other soluble protein (*N*_S_).

The amount of *N*_C_ determines absorption of photosynthetically active light in the leaf. Based on the equation of Evans (1993) for relating absorption to whole-leaf chlorophyll, the amount of *I*_abs_ in Equation 1 can be calculated by (Medlyn 1996):

$$I_{\text{abs}}=\frac{a_{\text{c}}N_{\text{C}}}{a_{\text{c}}N_{\text{C}}+b_{\text{c}}}I_{\text{inc}}$$ (2)

where *I*_inc_ is the incident light fluxes, and *a*_c_ and *b*_c_ are empirical coefficients (Table 2). Similar to most studies that use the FvCB model, Equation 2 implicitly assumes the absence of a gradient in chloroplast density or in the absorbed light intensity among different layers through the leaf. This assumption is valid only for relatively thin leaves (Farquhar *et al.*, 1980).

**Table 2.** Indicative values of coefficients used in the model

Coefficient	Equation	Unit	Value	Reference
*a*_c_	2	mmol Chl mol^−1^ N	25	Evans (1993); Medlyn (1996)
*a*_J_	3	μmol e^−^ mol^−1^ N s^−1^	15870	Medlyn (1996)
*b*_c_	2	mmol Chl m^−2^	0.076	Evans (1993); Medlyn (1996)
*D*_J_	A7	J mol^−1^	200000	Harley *et al.* (1992)
*E*	A6	J mol^−1^	65330 (for *V*_c,max_)	Bernacchi *et al.* (2002)
			80990 (for *K*_mC_)	Bernacchi *et al.* (2002)
			23720 (for *K*_mO_)	Bernacchi *et al.* (2002)
			24460 (for Γ*or for γ*)	Bernacchi *et al.* (2002)
			46390 (for *R*_d_)	Bernacchi *et al.* (2002)
*E*_Jmax_	A7	J mol^−1^	69100	Harley *et al.* (1992)
*f*_cyc_	A5	-	0.05	Yin *et al.* (2006)
*k*_s_	5	mol N s (μmol e^−^)^−1^	1.25 × 10^–4^	Medlyn (1996)
*K*_C25_	4	g CO_2_ g^−1^ Rubisco s^−1^	1.6 × 10^–3^	Farquhar *et al.* (1980)
*K*_mC25_	A6	μmol mol^−1^	291	Cousins *et al.* (2010)
*K*_mO25_	A6	mmol mol^−1^	194	Cousins *et al.* (2010)
*S*_c/o25_	for γ_*25_	mmol μmol^−1^	3.022	Cousins *et al.* (2010)
*S*_J_	A7	J K^−1^ mol^−1^	650	Harley *et al.* (1992)
*T*_opt_	A8	°C	22.5	Estimated from Yin *et al.* (2014)
ν	4	g Rubisco mol^−1^ N	87.72	Hikosaka and Terashima (1996)
τ	9	min	40 or variable	Kirschbaum *et al.* (1998)
Φ_2LL,max_	A8	mol e^−^ mol^−1^ photon	0.78	Estimated from Yin *et al.* (2014)
Φ_2LL_/Φ_1LL_	A5	–	0.85	Kingston-Smith *et al.* (1999)
Ω	A8	°C	36.5	Estimated from Yin *et al.* (2014)

*N*_T_ collectively refers to the amount of *N*_photo_ invested in all the components related to electron transport, including PSI and PSII reaction centres, cytochrome *b*_6_*f* (Cyt*f*), and other complexes. Note that although PSI and PSII have dual functions, dealing with both light harvesting and electron transport, nitrogen for the chlorophyll in the antennae systems of PSI and PSII is attributed to *N*_C_, and is not part of *N*_T_. This simplifies the calculation of *J*_max25_, being proportional to *N*_T_ only:

$$J_{\text{max}}=a_{\text{J}}N_{\text{T}}$$ (3)

where coefficient *a*_J_ is the proportion factor.

Assuming that Rubisco is fully activated *in vivo*, the maximum carboxylation velocity at the reference temperature (25 °C) is given by:

$$V_{\text{c},\text{max25}}=\text{1}0^{\text{6}}\nu K_{\text{C25}}N_{\text{R}}/\text{44}$$ (4)

where ν is the conversion coefficient from mol *N*_R_ to g Rubisco, *K*_C25_ is the specific activity of Rubisco at 25 °C, and 44 is the molecular weight of CO_2_.

Activities of the Calvin cycle enzymes, other than Rubisco, are not primary limiting factors of photosynthesis (Farquhar *et al.*, 1980), except for a possible limitation due to triose phosphate utilization (Sharkey, 1985). However, Medlyn (1996) discussed that the likely impact of triose phosphate metabolism on the determination of the nitrogen partitioning is small. It is therefore assumed that the amount of *N*_S_ is just sufficient to support the maximum rate of electron transport, *J*_max_ (Medlyn 1996; Evans and Poorter, 2001):

$$N_{\text{S}}=k_{\text{s}}J_{\text{max}}$$ (5)

where *k*_s_ is the proportion constant.

### Optimization procedure

Our objective is to determine the optimum distribution of photosynthetic nitrogen (*N*_photo_) among *N*_C_, *N*_T_, *N*_R_, and *N*_S_, such that leaf photosynthesis (*A*), calculated by Equation A1 in Supplementary Appendix A, is maximal for a given combination of incoming light, chloroplast [CO_2_], leaf temperature, and leaf nitrogen content. Based on Equation A1 and Equation 1, achieving this optimization is straightforward: the maximal leaf photosynthesis requires a nitrogen distribution over light-harvesting compounds, the electron transport complex, and Rubisco in such a way that:

$$V_{\text{c}}=V_{\text{j}}$$ (6)

$$J_{\text{max}}=\text{a}I_{\text{abs}}$$ (7)

The rationale for these requirements is that as long as they are not equal, nitrogen has not yet reached the optimum distribution since improvement of *A* would still be possible by redistribution. From Equations 5, 6, and 7, and a further equation

$$N_{\text{C}}+N_{\text{T}}+N_{\text{R}}+N_{\text{S}}=N_{\text{photo}}$$ (8)

the optimum value for *N*_C_, *N*_T_, *N*_R_, and *N*_S_ can be solved analytically, as given in Supplementary Appendix B. This is the unique solution with biologically realistic values; the other mathematical solution can lead to a negative value for *N*_C_.

The above procedure gives the nitrogen distribution that maximizes photosynthesis at a given value of *N*_photo_. To express model results in terms of *N*_leaf_, the relationship between *N*_photo_ and *N*_leaf_ has to be specified. The percentage of *N*_leaf_ allocated as *N*_photo_ can vary between 50% and 80% (Hikosaka and Terashima, 1995). For simplicity, we used roughly average relationships of: *N*_photo_=0.65*N*_leafE_ and *N*_leafE_=*N*_leaf_–0.02 where *N*_leafE_ is leaf nitrogen effective for physiological activities (Sinclair and Horie, 1989) assuming a base leaf nitrogen of 0.02 mol N m^−2^. The difference between *N*_leafE_ and *N*_photo_ refers to some inorganic nitrogen as well as the organic nitrogen used for processes other than photosynthesis.

### Modelling analysis

The above optimum nitrogen partitioning solution was applied to analyse several experiments reported by Yamori *et al.* (2005, 2010, 2011), where *N*_leaf_, net leaf photosynthesis (*A*), and photosynthetic protein components were measured for plants that were assumed to have acclimated fully to growth environments.

We assessed to what extent the optimum nitrogen partitioning could explain observed changes of the FvCB parameters with varying growth environment. As acclimation to temperature has been most studied in the literature (e.g. Yamasaki *et al.*, 2002; Yamori *et al.*, 2005; Hikosaka *et al.*, 2006; Kattge and Knorr, 2007; Stinziano *et al.*, 2018), we took temperature as the growth environment factor for this analysis. We used the FvCB model coupled with our optimum nitrogen partitioning algorithms to generate a set of photosynthetic CO_2_–response curves for plants grown at 15, 20, 25, 30, and 35 °C, respectively. For each growth temperature, CO_2_–response curves were generated for seven measurement leaf temperatures (10, 15, 20, 25, 30, 35, and 40 °C), all at *I*_inc_ of 1500 μmol m^−2^ s^−1^, and for green leaves (*N*_leaf_=0.15 mol m^−2^), mimicking a common measurement protocol for estimating parameter values of the FvCB model. The generated data were then fit to estimate *V*_c,max25_, *J*_max25_ (represented as *J*_25_ at the *I*_inc_ of 1500 μmol m^−2^ s^−1^), *E*_Vc,max_, and *E*_Jmax_ of each growth temperature, while leaving other parameters at their default values as shown in Table 2.

To simulate leaf photosynthesis of plants grown under dynamically fluctuating environments, all *N*_C_, *N*_T_, *N*_R_, and *N*_S_ are expressed as state variables and a change of their state with time (*t*) is modelled using the first-order kinetics:

$$\frac{\text{d}N_{\text{i}}}{\text{d}t}=(N_{\text{i,opt}}-N_{\text{i}})/\tau$$ (9)

where *N*_i_ represents *N*_C_, or *N*_T_, or *N*_R_, or *N*_S_, *N*_i,opt_ represents the optimum value of these variables as calculated for a given growth condition, and τ is a time constant (τ>0). The value of τ may differ for different components, but we assume that it is the same for these components so that the sum of simulated *N*_C_, *N*_T_, *N*_R_, and *N*_S_ is always equal to *N*_photo_. This approach does not consider any metabolic cost of acclimation, which appears to be minor and hard to quantify (Athanasiou *et al.*, 2010). We set τ to the value for the Rubisco activity, ~2400 s or 40 min (Kirschbaum *et al.*, 1998). Then actual leaf photosynthetic rates at any given set of environmental conditions can be calculated from the modified FvCB model where photosynthetic absorptance was conditional on the modelled values for *N*_C_ (Equation 2), *V*_c,max25_ on the value for *N*_R_ (Equation 4), and *J*_max_ on the value of *N*_T_ and *N*_S_ (Equations 3 and 5).

The model was run for wheat for a period of ~50 d, using actual weather data of every 10 min in an experiment under field conditions (Cai *et al.* 2016), where the average daylength was ~12.2 h. According to the principles discussed by Penning de Vries and van Laar (1982), a time step of 10 min for dynamic simulation was short enough for the process where the characteristic time constant is 40 min (note that the time step has to be ≤τ; otherwise, simulation with Equation 9 may yield meaningless values such as the oscillating pattern; Penning de Vries and van Laar, 1982). So, the total time steps of our simulation period were ~3650. The modelled ‘acclimating’ leaf photosynthetic rates from using Equation 9 were compared with the predictions by the version of the model without acclimation (‘non-acclimating’), where nitrogen partitioning was static, being fixed to the optimum values for the average environmental conditions of the time steps covered by simulation. The initial values of *N*_C_, *N*_T_, *N*_R_, and *N*_S_ for dynamic simulation were set to be the same as their values for the ‘non-acclimating’ simulation. Given the uncertainty of τ (Sassenrath-Cole and Pearcy, 1994; Kirschbaum *et al.*, 1998), several values of τ were used to implement the ‘acclimating’ version of the model. A direct comparison of this ‘acclimating’ model with the original steady-state FvCB model is not useful as many of the input parameters and their values differ between the two models.

For all above analyses, as leaf day respiration rate (*R*_d_) is not part of the optimum solutions (see the Discussion), we set *R*_d_ at 25 °C to be 0.01*V*_c,max25_, which is commonly observed (Harley *et al.*, 1992; Yamori *et al.*, 2005; Silva-Pérez *et al.*, 2017; Cai *et al.*, 2018) and used for general prediction (Medlyn *et al.*, 2002; Yin and Struik, 2017).

## Results

### Illustration of optimization results

Using the above algorithms with values of input parameters (Table 2), we calculated the optimum *N*_photo_ partitioning among the four pools in response to four variables: *I*_inc_, *N*_leaf_, *C*_c_, and leaf temperature. Values of some parameters in Table 2, although widely used, are only indicative given their reported variations among species and growth environments (e.g. Silva-Pérez *et al.*, 2017). They are used here merely to illustrate how the nitrogen partitioning varies qualitatively in response to growth environment.

**Fig. 1.**
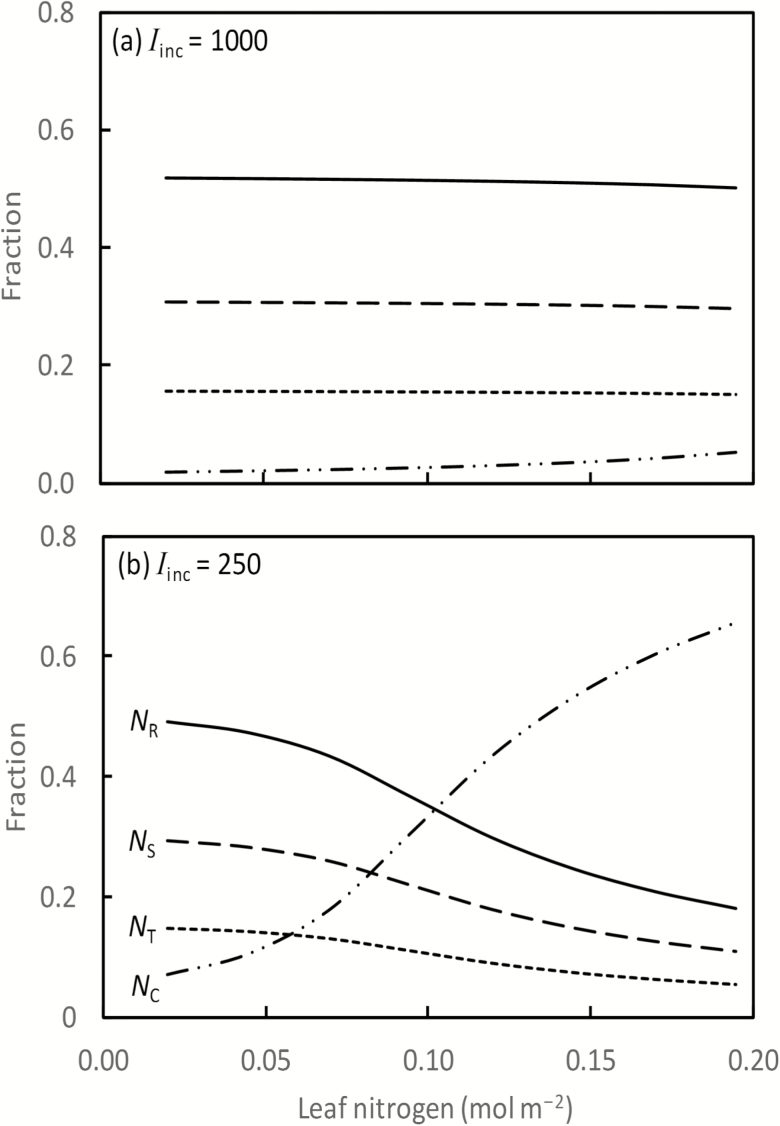
Predicted optimum partitioning of *N*_photo_ among *N*_C_, *N*_R_, *N*_T_, and *N*_S_, as affected by *N*_leaf_, at two levels of *I*_inc_ (µmol m^–2^ s^–1^). In this case, *C*_c_ = 250 µmol mol^−1^, leaf temperature=25 °C.

The optimum partitioning pattern versus *N*_leaf_ was calculated at various levels of *I*_inc_, and the results for *I*_inc_ at 1000 μmol m^−2^ s^−1^ and 250 μmol m^−2^ s^−1^ are given in Fig. 1. The resultant leaf photosynthesis when the optimum partitioning was reached in response to *I*_inc_ and *N*_leaf_ is illustrated in Supplementary Fig. S1. While a non-linearity was not explicitly assumed, our model did predict a non-linear response of leaf photosynthesis to both *I*_inc_ and *N*_leaf_ when partitioning was at the optimum. Specifically, the response to either *I*_inc_ or *N*_leaf_ was a non-rectangular hyperbola (Supplementary Appendix C). However, the curvature of predicted responses to *N*_leaf_ was weak under high light conditions (Supplementary Fig. S1b). The decrease in curvature with increasing light has also been observed experimentally (e.g. Makino *et al.*, 1997).

At high light levels (1000 μmol m^−2^ s^−1^), the optimum partitioning was hardly affected by the variation of *N*_leaf_ (Fig. 1a). At a low *I*_inc_ (250 μmol m^−2^ s^−1^), the nitrogen partitioning became more dependent on *N*_leaf_ itself (Fig. 1b). With low *I*_inc_ and increasing *N*_leaf_ levels, the optimum partitioning required *N*_photo_ increasingly invested preferentially in *N*_C_, accompanied by a reducing investment in *N*_R_, *N*_T_, and *N*_S_.

Relationships between relative fractions of *N*_C_, *N*_R_, *N*_T_, and *N*_S_ predicted by the model were linear, and these relationships were not altered by the level of *I*_inc_ or *N*_leaf_ (Supplementary Fig. S2a). At the same leaf temperature and the same *C*_c_, the linearity between *N*_T_ and *N*_S_ can be expected from Equations 3 and 4, and that between *N*_R_ and *N*_T_ can be expected from Equations B3 and B4 in Supplementary Appendix B. Since the total of *N*_C_, *N*_R_, *N*_T_, and *N*_S_ was a fixed value at a given *N*_leaf_, a linearity between *N*_C_ and the other three pools was also expected. Overall, the fraction partitioned to *N*_C_ was most variable (Supplemtnary Fig. S2).

For an elevated CO_2_ condition (*C*_c_=500 μmol mol^−1^), a pattern similar to that given in Fig. 1 was obtained (results not shown) and, again, linear relationships between relative fractions of *N*_C_, *N*_R_, *N*_T_, and *N*_S_ were predicted (Supplemtnary Fig. S2b). However, the relative fraction to *N*_R_ was reduced, and those to *N*_C_, *N*_T_, and *N*_S_ were increased, relative to the fractions obtained for the default CO_2_ condition. This predicted effect of CO_2_ on the *N*_photo_ partitioning can be seen from changes in coefficients in linear relationships between relative fractions of *N*_C_, *N*_R_, *N*_T_, and *N*_S_, through comparison with those at *C*_c_=250 μmol mol^−1^ (Supplementary Fig. S2).

**Fig. 2.**
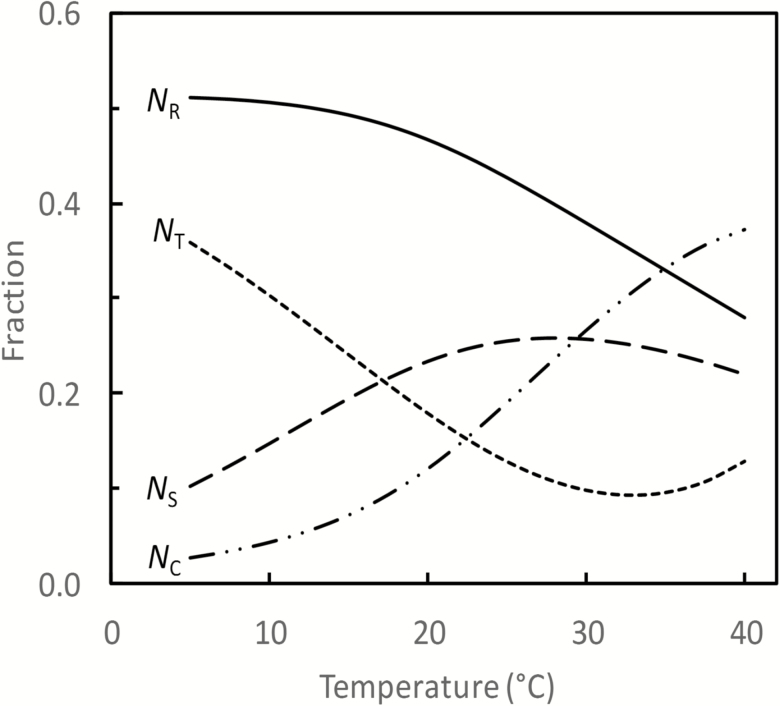
Predicted optimum partitioning of *N*_photo_ among *N*_C_, *N*_R_, *N*_T_, and *N*_S_, as affected by leaf temperature, under the condition that *I*_inc_ = 500 µmol m^–2^ s^–1^, *C*_c_ = 250 µmol mol^–1^, and *N*_leaf_ = 0.15 mol m^–2^.

We also examined the effect of leaf temperature on the optimum partitioning by varying the temperature from 5 °C to 40 °C. Figure 2 shows the result of optimization under the condition that *I*_inc_=500 μmol m^−2^ s^−1^, *C*_c_=250 μmol mol^−1^, and *N*_leaf_=0.15 mol m^−2^. For other conditions, the predicted trend was similar. With increasing temperature, the fraction to *N*_R_ decreased continuously, but more rapidly so at higher temperature ranges; the fraction to *N*_C_ or *N*_S_ increased generally, but *N*_S_ reached an optimum at ~28 °C. The fraction to *N*_T_ first declined, followed by an increase beyond a certain high temperature. This pattern for *N*_T_ was due to the assumed optimum response of *J*_max_ to temperature (the peaked Arrhenius equation, Equation A7 in Supplementary Appendix A). As expected, the linear relationships between relative fractions of *N*_C_, *N*_R_, *N*_T_, and *N*_S_ as shown in Supplementary Fig. S2 did not exist when leaf temperature varied (results not shown). The result of optimization by varying temperature showed interactive effects of temperature with other variables (*I*_inc_, *C*_c_, and *N*_leaf_) on leaf photosynthesis (SupplementaryFig. S3). First, responses of photosynthesis to a change in temperature were stronger when other variables were closer to the favourable level. Secondly, the optimum temperature for photosynthesis varied with other variables. The optimum temperature increased with increasing *I*_inc_ or *C*_c_ (Supplementary Fig. S3a, c), and declined with increasing leaf nitrogen (Supplementary Fig. S3b).

### Comparison of optimized nitrogen partitioning with acclimation experiments

**Fig. 3.**
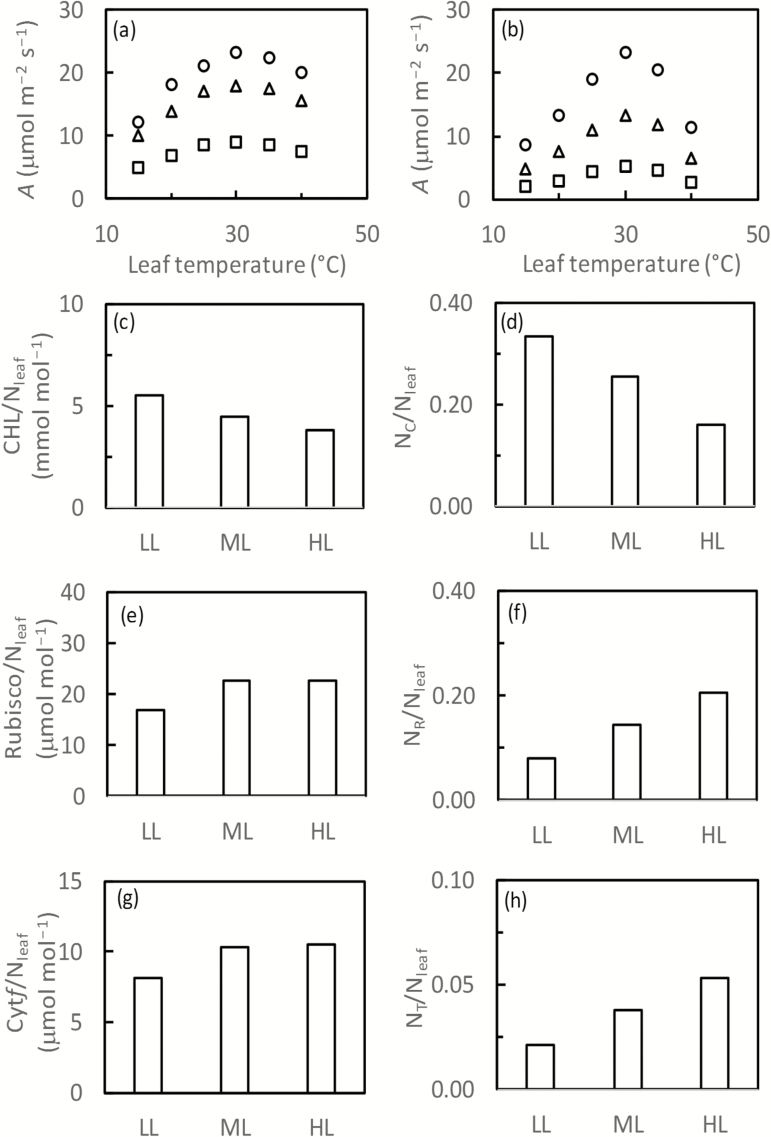
Observed (a) and simulated (b) net CO_2_ assimilation rate (*A*, at 1500 µmol m^–2^ s^–1^ irradiance and ambient CO_2_ level) in response to measurement temperature, and observed amounts of photosynthetic proteins per unit *N*_leaf_ (c, e, g) versus predicted nitrogen in equivalent protein compounds per unit *N*_leaf_ (d, f, h) in leaves of tobacco plants grown in low light (LL, 100 µmol m^–2^ s^–1^), medium light (ML, 250 µmol m^–2^ s^–1^), and high light (HL, 450 µmol m^–2^ s^–1^). Other growth conditions: day/night temperature=30/25 °C, *N*_leaf_=0.0809, 0.1137, and 0.1409 mol m^–2^ s^–1^ for LL, ML, and HL, respectively (experimental data from Yamori *et al.*, 2010). In (a) and (b), squares, triangles, and circles represent LL, ML, and HL leaves, respectively.

We compared our modelled optimum nitrogen partitioning with actual experimental measurements (Fig. 3; Supplementary Figs S4, S5). For the data set of Yamori *et al.* (2010), the modelled temperature response curves for tobacco plants grown in three different light levels agreed roughly with the measured curves (Fig. 3a, b). In line with earlier predictions shown in Fig. 1, the modelled *N*_C_:*N*_leaf_ ratio decreased, while the *N*_R_:*N*_leaf_ and *N*_T_:*N*_leaf_ ratios increased, with increasing light levels during growth. Such trends qualitatively agreed with the measured CHL:*N*_leaf_, Rubisco:*N*_leaf_, and Cyt*f*:*N*_leaf_ ratios, respectively, in response to the light levels during growth (Fig. 3c–h), although the measured Rubisco:*N*_leaf_ and Cyt*f*:*N*_leaf_ ratios did not differ significantly between medium and high light levels (Fig. 3e, g).

For the data set of Yamori *et al.* (2005) for spinach plants grown in high (HT) and low (LT) temperatures, the modelled temperature response curves under the optimum nitrogen partitioning differed from the measured curves (Supplementary Fig. S4a, b). The LT plants had a much higher *N*_leaf_ (0.1269 mol m^−2^) than the HT plants (0.0811 mol m^−2^), resulting in a constantly higher *A* for LT than for HT plants across measurement temperatures (Supplementary Fig. S4b). However, the model predicted a lower optimum temperature for plants grown in the LT than in the HT. The modelled *N*_C_:*N*_leaf_ ratio hardly differed between HT and LT conditions, but the measured CHL:*N*_leaf_ ratio was slightly lower in the LT than in the HT condition (Supplementary Fig. S4c, d). The modelled *N*_R_:*N*_leaf_ and *N*_T_:*N*_leaf_ ratios were lower with HT than with LT, in line with the measured Rubisco:*N*_leaf_ and Cyt*f*:*N*_leaf_ ratios in response to the growth temperature (Supplementary Fig. S4e–h).

For the data set of Yamori *et al.* (2011) for plants of four species (wheat, rice, spinach, and tobacco) grown in low, medium, and high nitrogen conditions (LN, MN, and HN), we show the average results of four species in order to assess any impact of nitrogen (Supplementary Fig. S5). The modelled temperature response curves under the optimum nitrogen partitioning using the default parameters of Table 2 were somewhat lower than the measured curves (Supplementary Fig. S5a, b). The measured CHL:*N*_leaf_ ratio hardly changed, while the measured Rubisco:*N*_leaf_ ratio slightly increased and the measured Cyt*f*:*N*_leaf_ ratio slightly decreased, with increasing nitrogen supply (Supplementary Fig. S5c, e, g). In comparison, the modelled *N*_C_:*N*_leaf_ ratio increased slightly with increasing nitrogen level, while the modelled *N*_R_:*N*_leaf_ and *N*_T_:*N*_leaf_ ratios changed little among nitrogen environments (Supplementary Fig. S5d, f, h).

### Modelled changes in FvCB parameter values with growth temperature

**Fig. 4.**
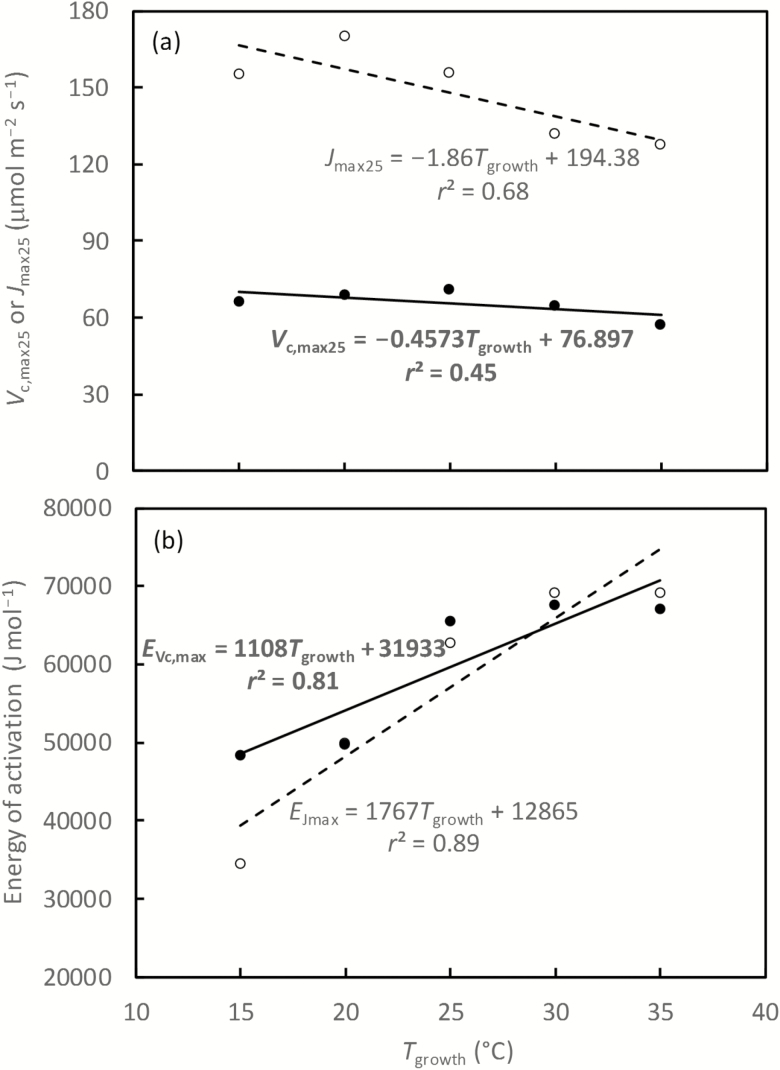
Estimated values of the FvCB model parameters plotted against growth temperature *T*_growth_: (a) *V*_c,max25_ (filled symbols and the solid line) and *J*_max25_ (open symbols and the dashed line), (b) *E*_Vc,max_ (filled symbols and the solid line), and *E*_Jmax_ (open symbols and the dashed line). See the text for details of the data that were generated for the analysis shown in this figure.

Using the generated *A*–*C*_c_ curves at different measurement temperatures as earlier described, values of *V*_c,max25_, *J*_max25_, *E*_Vc,max_, and *E*_Jmax_ of the standard FvCB model were simultaneously fitted for each growth temperature. The model fit improved with increasing growth temperature, with *R*^2^ of 0.66, 0.77, 0.93, 0.99, and 0.99 for growth temperatures of 15, 20, 25, 30, and 35 °C, respectively. Overall, estimated *V*_c,max25_ and *J*_max25_ decreased only slightly with increasing growth temperature (Fig. 4a). In contrast, estimated *E*_Vc,max_ and *E*_Jmax_ increased with increasing growth temperature, with *E*_Jmax_ increasing faster than *E*_Vc,max_ (Fig. 4b).

### **Comparison between simulated photosynthesis rates using** ‘**acclimating**’ **scenarios and the scenario without acclimation**

We ran the model, using Equation 9 to mimic dynamic acclimation. To visualize this process, the result of simulation when *I*_inc_ changed abruptly at a given time step between 1000 μmol m^−2^ s^−1^ and 300 μmol m^−2^ s^−1^ is shown in Supplementary Fig. S6 for three contrasting values of time constant τ. The difference in simulated *A* among three values of τ was greater when *A* was increasing than when it was decreasing (Supplementary Fig. S6).

**Fig. 5.**
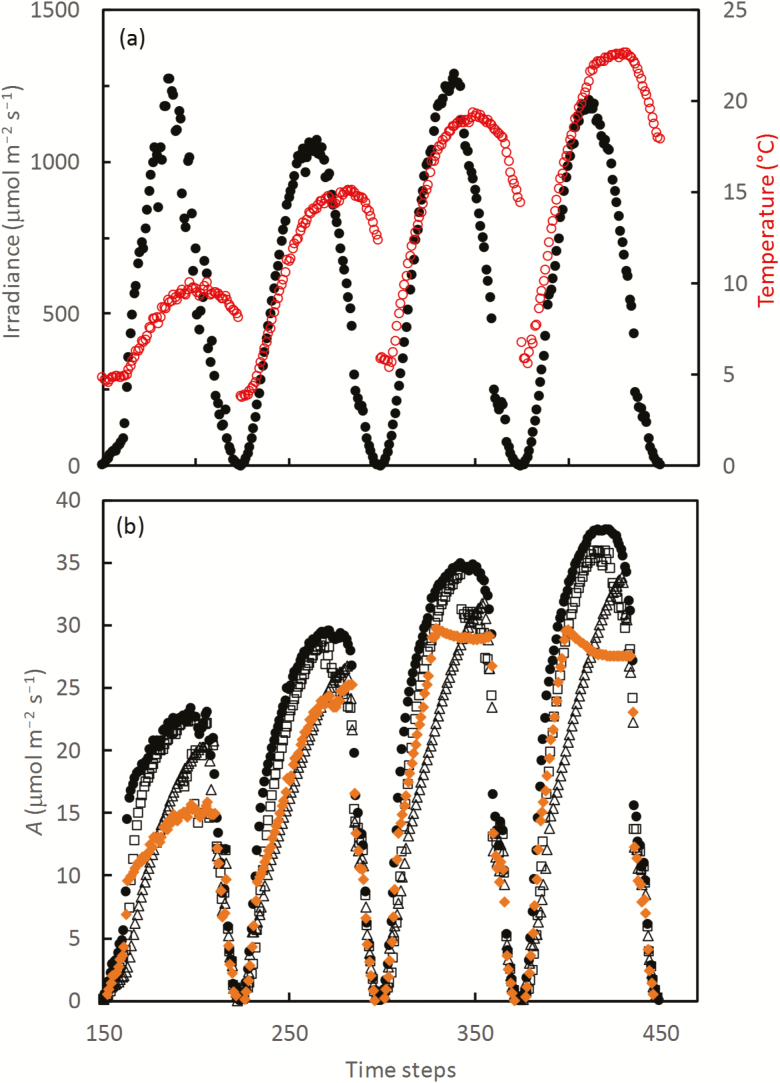
Daytime course for randomly selected four consecutive days, of (a) incoming irradiance (filled circles) and temperature (open circles), and (b) simulated net CO_2_ assimilation rate (*A*) under four simulation scenarios: (i) the optimum N partitioning (black circles); (ii) dynamic acclimation using Equation 9 where τ was set to its default value 40 min (open squares); (iii) dynamic acclimation using Equation 9 where τ was extended to 160 min (open triangles); and (iv) the case without acclimation where nitrogen partitioning was static, set to the optimum values for the season-long average environmental conditions (orange-coloured lozenges). The simulation time step was to 10 min, and the total number of time steps was ~3650, equivalent to the daytime period of ~50 d (see the text).

For a more realistic field condition, we implemented simulation for a time period of ~50 d (see the section ‘Modelling analysis’), for upper, middle, and bottom layers of leaves in a canopy, which were assumed to be exposed to 100, 50, and 15% of incoming irradiance levels. As such, we did not consider here any specific change in instantaneous irradiance for leaves as a result of diurnal sun angle, passing clouds, and sunflecks, which are often taken into account in other routines of general simulation models. The level of *N*_photo_ for these layers was assumed to scale with their exposed irradiance levels, being 0.10, 0.05, and 0.015 mol m^−2^, respectively. Irradiance and temperature for a period of four consecutive days, and the equivalent simulated *A* are shown in Fig. 5 for the upper leaves only, since the pattern was similar for the other two layers except for their lower absolute values of simulated *A* compared with the upper layer.

As expected, the simulated *A* assuming acclimation with the default time constant τ (40 min) was lower than the values of *A* under the optimum nitrogen partitioning (*A*_opt_) (Fig. 5). Increasing τ led the simulated *A* to deviate more from *A*_opt_, and the difference between *A* and *A*_opt_ was more significant when *A* was increasing than when *A* was decreasing within a day (Fig. 5).

The diurnal pattern of simulated *A* by the version of the model without acclimation (‘non-acclimating’), *A*_without_, differed from that using the versions of the model assuming acclimation (Fig. 5). The simulated *A*_without_ could reach a maximum value over certain hours around noon for the days when the incoming irradiance (e.g. the third and fourth days shown in Fig. 5) was high, whereas such a plateau was never simulated using the model versions assuming acclimation.

Daily integrals of *A*, *A*_opt_, and *A*_without_ can be calculated from their simulated instantaneous values. We plotted the *A*/*A*_without_ ratio against the daily *A*_opt_ for two values of τ in the upper leaves (Supplementary Fig. S7), where the variation in daily *A*_opt_ over the season indicated the day-to-day variation largely in solar radiation. The *A*/*A*_without_ ratio was close to 1.0 when *A*_opt_ was between 0.5 mol m^−2^ d^−1^ and 1.0 mol m^−2^ d^−1^, whereas the ratio tended to increase when *A*_opt_ was <0.5 mol m^−2^ d^−1^ (cloudy or rainy days) and/or >1.0 mol m^−2^ d^−1^ (sunny days). Also, a low τ increased the *A*/*A*_without_ ratio (Supplementary Fig. S7).

**Fig. 6.**
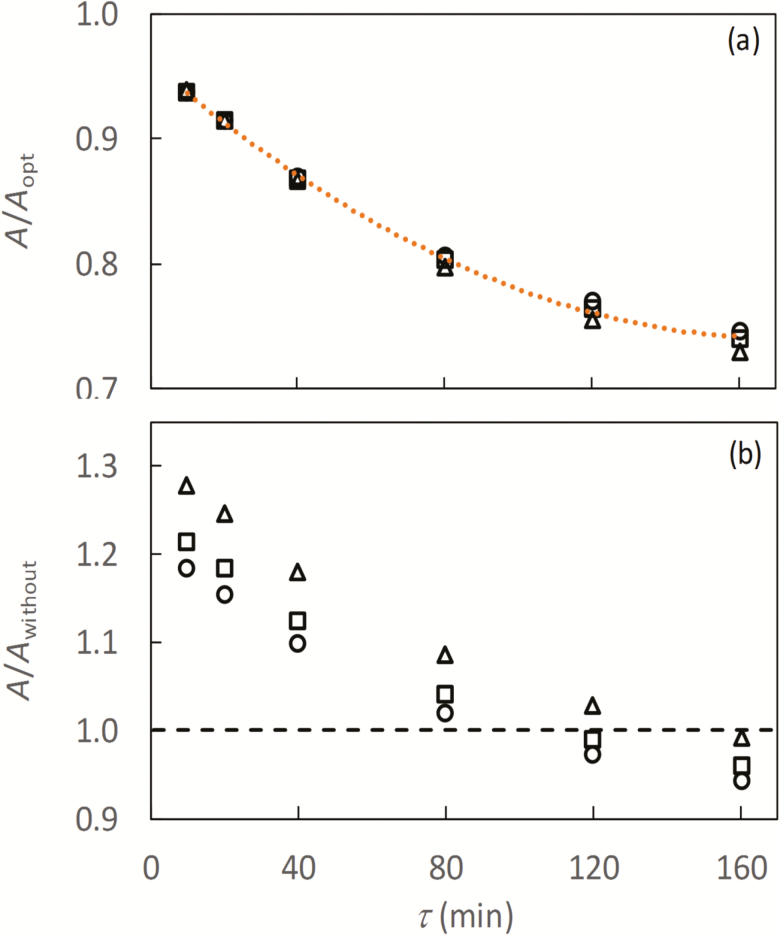
Ratios of simulated net CO_2_ assimilation rate *A* assuming dynamic acclimation using Equation 9 with different values of time constant τ, either to the simulated net CO_2_ assimilation rate assuming the optimum N partitioning, *A*_opt_ (a), or to the simulated net CO_2_ assimilation rate without acclimation assumed, *A*_without_ (b). Circles, squares, and triangles represent the results for upper, middle, and bottom leaves of a canopy, respectively. Symbols in this figure are drawn from the seasonal integral of simulated values.

Likewise, the ratios between *A* and *A*_opt_, and between *A* and *A*_without_ integrated over the season decreased with an increase in the value of τ (Fig. 6). The pattern did not differ much among the three layers of leaves in a canopy. However, the *A*/*A*_without_ ratio was somewhat higher in lower than in upper leaves (Fig. 6b), in line with the most significant variation in the nitrogen partitioning under low irradiance conditions (Fig. 1b). Overall, only when τ increased towards 120 min did the simulated ‘acclimating *A*’ approximately equal *A*_without_ (Fig. 6b).

## Discussion

### Optimization procedure

We present an analytical procedure to determine the optimum distribution of *N*_photo_ among photosynthetic protein complexes under a specific environmental condition, based on the FvCB model for C_3_ species. With this procedure, a computationally expensive numerical optimization procedure is avoided. This was achieved by modifying the original FvCB model that uses a non-rectangular hyperbolic equation, Equation A4 in Supplementary Appendix A, to describe the relationship between *J* and *I*_abs_ (Farquhar and Wong, 1984). This non-rectangular hyperbola requires an empirical parameter (θ) to define the curvature of the response, and its value appears to be determined arbitrarily in the literature; for example, 0.67 (Farquhar and Wong, 1984), 0.70 (Evans, 1993), 0.88 (Alonso *et al.*, 2009), 0.90 (Medlyn *et al.*, 2002), and 0.95 (Leuning, 1995). Medlyn (1996) has shown that the optimization result of the nitrogen partitioning is very sensitive to the value of this curvature parameter. Instead of using a non-linear model, we introduced Equation 1, which yielded an additional equation, Equation 7, needed to solve the optimum nitrogen partitioning.

One criticism of using Equation 1 could be that it predicts a constant electron transport efficiency of PSII over the range of irradiance within which *J*≤*J*_max_, whereas experimentally measured efficiencies of PSII decline almost linearly with irradiance (e.g. Harbinson *et al.*, 1990; Yin *et al.*, 2014). However, the observed decline of PSII efficiencies could be due to the feedback effect of the limitation set by Rubisco, which increasingly becomes rate limiting with an increase in irradiance. This reasoning is supported by an observation of a sharper decline of PSII efficiencies in nitrogen-starved than nitrogen-enriched leaves (Schapendonk *et al.*, 1999) or at a lower temperature (Kingston-Smith *et al.*, 1999), because photosynthesis in nitrogen-starved leaves or at low temperature is more Rubisco limited. Like the original FvCB model, our model does predict a decline in actual PSII efficiencies when *A* is limited by *V*_c_ rather than by *V*_j_. The use of Equation 1, rather than a non-rectangular hyperbolic relationship like Equation A4 in Supplementary Appendix A, is supported by an observation that the electron transport rate through PSII increases proportionally with irradiance to at least 1000 μmol m^−2^ s^−1^ if electron transport and CO_2_ fixation are uncoupled (Cheeseman and Lexa, 1996). Although Equation 1 is linear, our model did generate a hyperbolic non-linear photosynthetic response to irradiance (Supplementary Fig. S1a), suggesting that the nitrogen partitioning may also contribute a part to decreases of the measured PSII efficiency with increasing irradiance.

### Is photosynthetic nitrogen partitioned optimally?

It is necessary first to point out that the optimum nitrogen-partitioning solution depends on many model coefficients (Table 2) as well as on the coefficients for the relationship between *N*_photo_ and *N*_leaf_. Values of these coefficients are open to change, given the large variation between species in the nitrogen partitioning (Seemann *et al.*, 1987; Evans, 1989; Hikosaka, 1997). Thus, using only one set of default values of the coefficients contributed to discrepancies between modelled and measured values, either in photosynthetic rates or in nitrogen investments in photosynthetic proteins, for various species (Fig. 3; Supplementary Figs S4, S5). However, the choice of coefficient values does not change the overall trend of model prediction. Our results showed that the optimum nitrogen partitioning responds to environmental changes according to an induced imbalance between light energy absorbed through photochemistry and the energy utilized through stromal metabolism (Huner *et al.*, 1998). The model predicted a high investment of nitrogen in Rubisco under an environmental change that leads to an excessive energy supply relative to the energy demand by stromal metabolism (e.g. low temperature, high light, low nitrogen, low CO_2_). Conversely, more investment in the chlorophyll complex is needed when energy supply is limiting. This response was shown by our findings that fractions partitioned to *N*_C_ and *N*_R_ were most responsive to physiological or environmental changes (Fig. 1; Supplementary Fig. S2), in line with reports that nitrogen partitioning between light-harvesting and carboxylation complexes is nearly optimal (Evans, 1993; Hikosaka and Terashima 1996).

For example, partitioning to *N*_R_ was predicted to decrease in response to an elevated [CO_2_] (Supplementary Fig. S2). Substantial experimental evidence (e.g. Schapendonk *et al.*, 2000) has indicated a decline of leaf Rubisco content when plants are grown under elevated CO_2_ conditions, which may confirm our optimization result. However, this decline of Rubisco may be a result of a decrease in *N*_leaf_ (e.g. Geiger *et al.*, 1999), rather than a reallocation of nitrogen within a leaf. Medlyn (1996) predicted that under a doubling CO_2_ concentration from its ambient level, electron transport capacity relative to Rubisco carboxylation velocity (the *J*_max25_:*V*_c,max25_ ratio) should increase by 40%, which is in good agreement with our prediction (Supplementary Fig. S2). Since at elevated CO_2_, the efficiency of CO_2_ fixation by Rubisco is increased and so less of this enzyme is needed, Sage (1994) indicated that to use nitrogen optimally, the Rubisco content should be decreased and the nitrogen thus freed should be re-allocated to other limiting processes. However, experimental data do not always support this prediction (e.g. Akita *et al.*, 2012). Furthermore, our model also showed that the increase in the *J*_max25_:*V*_c,max25_ ratio in response to the elevated CO_2_ became smaller at a lower temperature (results not shown), suggesting a strong interaction between CO_2_ and temperature.

Interactions between these environmental variables can be shown, to some extent, even from the steady-state FvCB model. For example, the model predicts increases in the optimum temperature with rising CO_2_ or irradiance (Farquhar *et al.*, 1980), and the importance of these shifts in the temperature optimum in predicting impacts of climate changes on ecosystems has often been emphasized (e.g. Long 1991). Here we show changes in the optimum temperature in response not only to CO_2_ and irradiance but also to plant nitrogen status (Supplementary Fig. S3). The predicted decline of the optimum temperature with increasing *N*_leaf_ is supported by data of Walcroft *et al.* (1997), which illustrated a higher photosynthetic rate at 25 °C than at 30 °C for plants at high nitrogen, but a lower rate at 25 °C than at 30 °C for those at low nitrogen. However, Sage and Pearcy (1987) showed little apparent change in the optimum temperature with *N*_leaf_.

Our optimization results generally agree well with previous predictions (Friend, 1991; Hikosaka and Terashima, 1995; Medlyn, 1996) and experimental observations (Seemann *et al*., 1987; Evans, 1989; Makino *et al.*, 1994, 1997; Warren and Adams, 2001). The exception is the change in the predicted fraction to *N*_R_ under increasing *N*_leaf_ conditions; our model predicted a decrease in the fraction to *N*_R_ with an increase in *N*_leaf_ (Fig. 1b), the direction opposite to the reports of Friend (1991) and Hikosaka and Terashima (1995). However, our model also indicated that the decreasing partitioning to *N*_R_ under increasing *N*_leaf_ only became apparent at a low irradiance level (Fig. 1) and, when irradiance was moderately high (550 μmol m^−2^ s^−1^), the *N*_R_/*N*_leaf_ ratio hardly varied with the nitrogen environment (Supplementary Fig. S5f). Experimental evidence of Makino *et al.* (1994, 1997) showed that the ratio of Rubisco to *N*_leaf_ beyond a certain base value is constant (independent of light, temperature, and *N*_leaf_). Yamori *et al.* (2011) showed that the Rubisco/*N*_leaf_ ratio increased with increasing nitrogen supply (Supplementary Fig. S5e). Warren and Adams (2001) also found a consistent overinvestment in Rubisco. Medlyn (1996) hypothesized that leaves tend to maintain high Rubisco levels in order to take advantage of any high light periods because the response of Rubisco-limited photosynthesis to increasing Rubisco is stronger than the response of light-limited photosynthesis to increasing chlorophyll. An alternative explanation would be that Rubisco is not fully activated and its specific activity is lower *in vivo* (Evans, 1989). Thus, our prediction, using the *in vitro* measured *K*_C25_ value (Farquhar *et al.*, 1980), only indicates the minimum quantity of nitrogen that may be present in Rubisco.

### Can the optimum nitrogen partitioning be used to predict photosynthetic acclimation?

Our model based on the optimum nitrogen partitioning can explain, at least to a considerable extent, increases in the optimum temperature with increasing growth temperature as reported by, for example, Yamasaki *et al.* (2002) and Yamori *et al.* (2005). Such changes can be obtained from the modelled increase in *E*_Vc,max_ and *E*_Jmax_ with increasing growth temperature (Fig. 4b). Following the previous meta-analysis procedure (e.g. Hikosaka *et al.*, 2006), we calculated a linear relationship between *E*_Vc,max_ or *E*_Jmax_ and growth temperature (Fig. 4), although the real relationship may be more complex. Our intercept and slope values for *E*_Vc,max_ were 31 933 J mol^−1^ and 1108 J mol^−1^ °C^−1^ (Fig. 4b), which are remarkably similar to 34 100 J mol^−1^ and 1010 J mol^−1^ °C^−1^, respectively, the values of Hikosaka *et al.* (2006) from their meta-analysis. We did not find equivalent quantitative information in the literature in support of our modelled intercept and slope values for *E*_Jmax_ shown in Fig. 4b, but the optimum temperature for *J*_max_ increased with growth temperature (Kattge and Knorr, 2007), suggesting that *E*_Jmax_ may increase with increasing growth temperature as well (Hikosaka *et al.*, 2006). Thus, relationships used in ecosystem models for accommodating photosynthetic thermal acclimation (e.g. Stinziano *et al.*, 2018), which were based on empirical equations like those of Hikosaka *et al.* (2006) and Kattge and Knorr (2007), can be the emergent properties of our optimum nitrogen partitioning model.

Such empirical relationships only reflect the consequence of acclimation, but do not model the dynamics of acclimation as a process *per se*, especially not for acclimation to rapidly varying field environmental conditions. We therefore incorporated algorithms for dynamic adjustment of the nitrogen content of a compound towards its optimum level, to predict leaf photosynthesis in rapidly varying environments. For that, we assumed the first-order kinetics, Equation 9, using a characteristic time constant (τ).

The diurnal course of simulated leaf photosynthesis, *A*, differed from the course generated by the steady-state version of the model without acclimation assumed, *A*_without_ (Fig. 5). A major feature of the simulated *A*_without_ is that a threshold value was predicted at time steps around noon, which varied little with further increases in irradiance on sunny days, whereas this threshold value was never simulated using the model version of the first-order kinetics. These results imply that the conventional steady-state FvCB model may not suffice for the temporally explicit situations where instantaneous rates of leaf photosynthesis are needed to be predicted accurately. However, daily integrated values of the simulated photosynthetic rate did not always significantly differ between the models with and without acclimation (Supplementary Fig. S7), depending on daily weather conditions and the time constant for simulation. This implies the importance of choosing appropriate environmental ranges in parameterizing the model if the steady-state version is used to simulate photosynthesis under varying conditions. For a further higher temporal scale, seasonally integrated values of the simulated photosynthetic rate, the difference between the models with and without acclimation could totally depend on the time constant τ (Fig. 6b).

The value for τ is uncertain and it may vary with species. As our model only considers nitrogen partitioning, τ may also lump the value for other processes that might contribute to the biochemical aspect of acclimation. In our analysis, we used the same value for τ for different nitrogen components, and its default value was set to 40 min according to the value for Rubisco-related activity (Kirschbaum *et al.*, 1998). However, τ for acclimation may depend on growth temperature (Maeva Baumont, INRA, France, personal communication), and the time for the protein turnover may differ among individual compounds (Huner *et al.*, 1998). A possible higher value of τ for Rubisco, relative to that for other components (Kirschbaum *et al.*, 1998) may contribute to the aforementioned overinvestment in Rubisco. The actual nitrogen partitioning in nature may never be at the optimum, resulting in an imbalance between energy supply and demand in chloroplasts (Huner *et al.*, 1998). As such, plants may always have to engage various photoprotective strategies to minimize photoinhibition (Ort and Baker, 2002).

In addition, our model ignores (i) stomatal conductance; (ii) mesophyll conductance; (iii) triose phosphate utilization limitation; and (iv) the partitioning of *N*_leafE_ between *N*_photo_ and other physiological nitrogen complexes, which all may play a part in photosynthetic acclimation. Also, in our analysis, *R*_d25_ is assumed to scale with *V*_c,max25_, which qualitatively agrees with the observation that *R*_d25_ per unit *N*_leafE_ is lower for plants grown at high temperature than for those grown at low temperature (data of Yamori *et al.*, 2005), and for plants grown under low light than for those grown under high light (data of Yamori *et al.*, 2010). However, temperature response of respiration may vary among growth conditions (Harley *et al.*, 1992; Walcroft *et al.*, 1997; Tjoelker *et al*., 2001; Yamori *et al.*, 2005; King *et al.*, 2006; Alonso *et al.*, 2009). There is also a possible response in the partitioning of *N*_leafE_ between *N*_photo_ and respiratory proteins, to both a daytime and night-time growth environment, which our model does not account for. More importantly, in the longer term, morphological acclimation, such as the variation of specific leaf area, can play a dominant role in determining photosynthetic acclimation (Evans and Poorter, 2001). It was probably due to this morphological acclimation that plants grown in different treatment environments have different values of *N*_leaf_ (Fig. 3; Supplementary Figs S4, S5). However, adding these parameters or processes would make it impossible to solve the nitrogen partitioning analytically, and morphological acclimation in particular would need to introduce other mechanisms to model. Nevertheless, since *N*_leaf_ is a physiological variable that is often simulated in general plant or crop models (e.g. Yin and Struik, 2017; Wu *et al.*, 2018), our methodology based on the optimum nitrogen partitioning can be incorporated into these models for simulating plant acclimation to varying environmental conditions.

## Supplementary data

Supplementary data are available at *JXB* online

Appendix A. Summary of the FvCB model for leaf photosynthesis.

Appendix B. Solution to the optimum partitioning of *N*_photo._

Appendix C. Model-generated responses of leaf photosynthesis to both *I*_inc_ and N_leaf._

Fig. S1. Model-generated leaf photosynthesis rate under the optimum nitrogen partitioning (*A*_opt_) in response to irradiance and leaf nitrogen content.

Fig. S2. Relationships between relative fractions of partitioning to *N*_C_, *N*_R_, *N*_T_, and *N*_S_ under two levels of CO_2_.

Fig. S3. Model-generated leaf photosynthesis rate under the optimum nitrogen partitioning, *A*_opt_, in response to leaf temperature.

Fig. S4. Observed and simulated temperature response of net CO_2_ assimilation rate, and observed amounts of photosynthetic proteins per unit *N*_leaf_ versus predicted nitrogen in equivalent protein compounds per unit *N*_leaf_ in leaves of spinach plants grown in low and high temperature (data from Yamori *et al.*, 2005).

Fig. S5. Observed and simulated temperature response of net CO_2_ assimilation rate, and observed amounts of photosynthetic proteins per unit *N*_leaf_ versus predicted nitrogen in equivalent protein compounds per unit *N*_leaf_ in leaves of four species grown in low, medium, and high nitrogen (data from Yamori *et al.*, 2011).

Fig. S6. Kinetics of net leaf photosynthesis *A* when incoming irradiance is abruptly changed between 1000 μmol m^−2^ s^−1^ and 300 μmol m^−2^ s^−1^ at the 20th time step of simulation.

Fig. S7. The ratio of daily photosynthetic rate simulated assuming acclimation to daily rate simulated without acclimation assumed, plotted against daily photosynthetic rate with the instantaneous optimum N partitioning over a period of ~50 d.

Supplementary Appendix S1-S3 and Figures S1-S7
