# Supplementary material for: Exploring the optimum nitrogen partitioning to predict the acclimation of C3 leaf photosynthesis to varying growth conditions
Source: J Exp Bot. 2018 Jul 25;70(9):2435–47. doi: 10.1093/jxb/ery277 (PMC6519419; doi:10.1093/jxb/ery277)
Supplement: Supplementary Appendix S1-S3 and Figures S1-S7 [file ery277_suppl_supplementary_appendix-s1-s3_figures-s1-s7.pdf]

Supplementary data

Supplementary Appendix A *Summary of the FvCB model for leaf photosynthesis*

The FvCB model describes the net rate of photosynthesis (A) as the minimum of the rate of carboxylation under the limitation of Rubisco activity (V_c) and the rate under the limitation of electron transport (V_j):

$$A = (1 - \Gamma_* / C_c) \cdot \min(V_c, V_j) - R_d \quad \text{(A1)}$$

where C_c is the chloroplast CO_2 concentration, R_d is day respiration, and Γ_* is the CO_2 compensation point in the absence of R_d and could be calculated as $O\gamma_*$ (O is the O_2 level in the chloroplast and γ_* is half of the inverse of Rubisco relative CO_2/O_2 specificity value $S_{c/o}$, i.e. $\gamma_* = 0.5/S_{c/o}$).

V_c is calculated using a C_c-dependent function, $V_{c(C_c)}$, as:

$$V_c = V_{c,max} V_{c(C_c)} = V_{c,max} \frac{C_c}{C_c + K_{mC}(1 + O / K_{mO})} \quad \text{(A2)}$$

where K_{mC} and K_{mO} are the Michaelis-Menten constant for CO_2 and O_2, respectively.

Calculation of V_j is given by:

$$V_j = J V_{j(C_c)} = J \frac{C_c}{4C_c + 8\Gamma_*} \quad \text{(A3)}$$

where J is the rate of whole-chain electron transport supporting CO_2-assimilation and photorespiration.

A non-rectangular hyperbolic equation was used to describe the dependence of J on chloroplast-absorbed photon flux, I_{abs}:

$$J = [\alpha I_{abs} + J_{max} - \sqrt{(\alpha I_{abs} + J_{max})^2 - 4\theta \alpha I_{abs} J_{max}}] / 2\,\theta \quad \text{(A4)}$$

where α is electron transport efficiency on the basis of irradiance absorbed by the two photosystems. In a general use of the FvCB model, α has been empirically set to constant (e.g. Harley et al. 1992; Alonso et al. 2009). According to Yin et al. (2004), α can be quantified theoretically by:

$$\alpha = \frac{\Phi_{2LL}(1 - f_{cyc})}{(1 - f_{cyc}) + \Phi_{2LL} / \Phi_{1LL}} \quad \text{(A5)}$$

where f_{cyc} is the fraction of total PSI electron flux that follows cyclic electron transport, and Φ_{2LL}/Φ_{1LL} is the PSII to PSI electron transport efficiency ratio under strictly limiting light conditions.

The temperature dependence of R_d and Rubisco kinetic parameters ($V_{c,max}$, γ_*, K_{mC} and K_{mO}) is described by an Arrhenius function normalised with respect to 25°C:

$$\text{Parameter} = \text{Parameter}_{25} \cdot \exp\left[\frac{(T - 25)E}{298R(T + 273)}\right] \quad \text{(A6)}$$

where E is the relevant activation energy for each of these parameters, R is the universal gas constant (Table 2).

A modified Arrhenius function is commonly used to describe the dependence of J_{max} on temperature, $J_{(T)}$, as:

$$J_{max} = J_{max\,25} J_{(T)} = \frac{J_{max\,25} \exp\{(T - 25)E_{Jmax} / [298R(T + 273)]\}}{1 + \exp\{[S_J(T + 273) - D_J]/[R(T + 273)]\}} \quad \text{(A7)}$$

where J_{max25} is J_{max} at the reference temperature 25°C, S_J is an entropy term, E_{Jmax} and D_J are the energies of activation and deactivation, respectively (Table 2). Some studies (e.g. Leuning et al. 1995; Kattge and Knorr 2007) also used eqn (A7) to describe the temperature dependence of $V_{c,max}$; however, we found no direct evidence, from either *in vivo* or *in vitro* measurements in the literature, that $V_{c,max}$ declines at high temperature up to 40°C. Therefore, we applied eqn (A6) to $V_{c,max}$.

Parameter Φ_{2LL} has been found to follow an optimum response to leaf temperature (Bernacchi et al. 2003; Yin et al. 2014) and this is modelled empirically by a normal-distribution equation:

$$\Phi_{2LL} = \Phi_{2LL,max} e^{-[(T - T_{opt})/\Omega]^2} \tag{A8}$$

where Ω is difference between the optimum temperature (T_{opt}) and the temperature at which Φ_{2LL} falls to e^{-1} of its maximum $\Phi_{2LL,max}$.

Supplementary Appendix B *Solution to the optimum partitioning of* N_{photo}

The optimum partitioning of N_{photo} among N_S, N_C, N_R and N_T can be obtained from a simultaneous solving of eqns (5-8) in the main text as:

$$N_S = \frac{b - \sqrt{b^2 - 4ac}}{2a} \tag{B1}$$

$$N_C = \frac{b_c N_S}{a_c [k_s \alpha I_{inc} - N_S]} \tag{B2}$$

$$N_R = \frac{44 a_J V_{j(C_c)} J_{(T)} (N_{photo} - N_S - N_C)}{10^6 \nu K_{C25} V_{c(T)} V_{c(C_c)} + 44 a_J V_{j(C_c)} J_{(T)}} \tag{B3}$$

$$N_T = N_{photo} - N_S - N_C - N_R \tag{B4}$$

where constants a, b and c in eqn (B1) are calculated by:

$$a = a_c (10^6 \nu K_{C25} V_{c(T)} V_{c(C_c)} + 44 a_J V_{j(C_c)} J_{(T)} + 10^6 \nu K_{C25} k_s a_J V_{c(T)} V_{c(C_c)} J_{(T)}) \tag{B5}$$

$$\begin{aligned} b = {} & a_c k_s \alpha I_{inc} (10^6 \nu K_{C25} V_{c(T)} V_{c(Cc)} + 44 a_J V_{j(C_c)} J_{(T)}) \\ & + 10^6 \nu K_{C25} a_c k_s a_J V_{c(T)} V_{c(C_c)} J_{(T)} N_{photo} \\ & + 10^6 \nu K_{C25} a_c k_s^2 a_J \alpha I_{inc} V_{c(T)} V_{c(C_c)} J_{(T)} \\ & + 10^6 \nu K_{C25} b_c k_s a_J V_{c(T)} V_{c(C_c)} J_{(T)} \end{aligned} \tag{B6}$$

$$c = 10^6 \nu K_{C25} a_c k_s^2 a_J \alpha I_{inc} V_{c(T)} V_{c(C_c)} J_{(T)} N_{Photo} \tag{B7}$$

where $V_{c(C_C)}$ and $V_{j(C_C)}$ are defined in eqns (A2) and (A3), respectively; $V_{c(T)}$ and $J_{(T)}$ are formulated from eqns (A6), and (A7), respectively, and α is calculated by eqn (A5).

Supplementary Appendix C *Model-generated responses of leaf photosynthesis to both I_{inc} and N_{leaf}*

If the partitioning of leaf nitrogen is at the optimum, leaf photosynthesis is proportional to I_{abs}:

$$A = \varepsilon I_{abs} = \varepsilon \frac{a_c N_C}{a_c N_C + b_c} I_{inc} \quad \text{(C1)}$$

where ε is solved from eqns (A3-A5). Substituting eqn (B2) into eqn (C1) and then simplifying the relation give:

$$A = \frac{\varepsilon N_S}{k_s \alpha} \quad \text{(C2)}$$

By substituting eqn (B1) into eqn (C2), it can be seen that responses of A to I_{inc} and N_{photo} (therefore, to N_{leaf} as well) are both in the form of non-rectangular hyperbola.

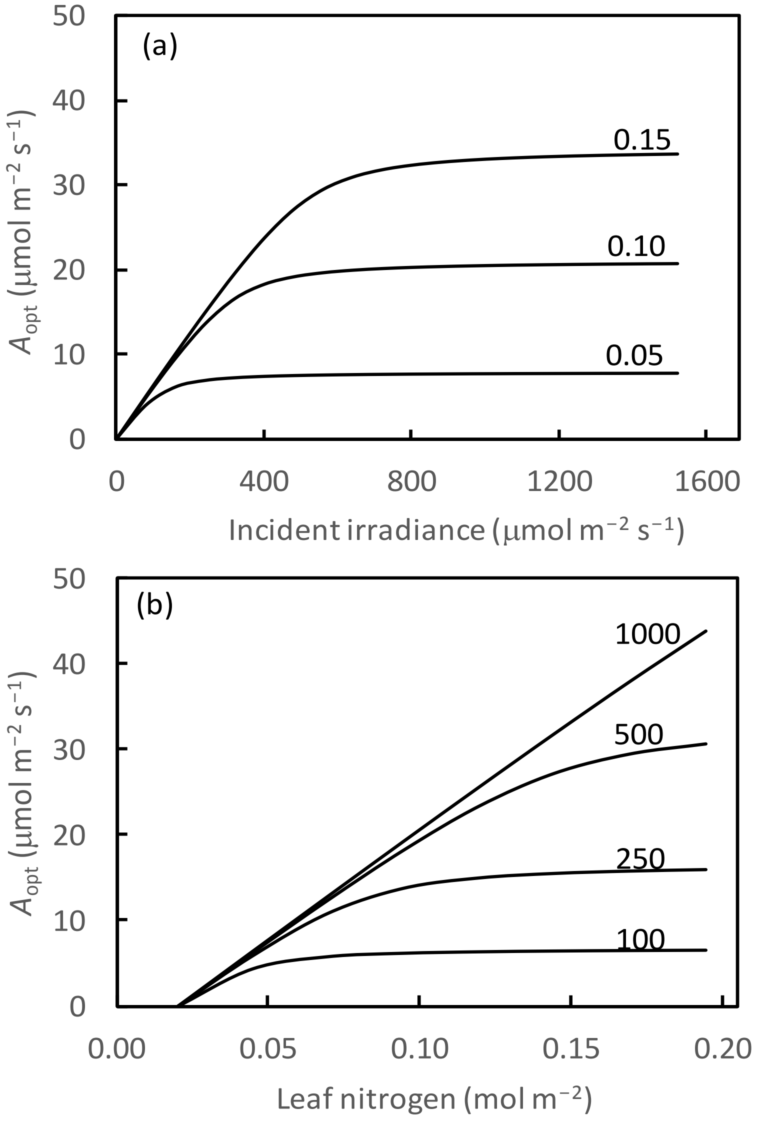

Fig. S1. Model-generated leaf photosynthesis rate under the optimum nitrogen partitioning (A_{opt}) in response to I_{inc} at three levels of N_{leaf} (mol m^{-2}) (a), and in response to N_{leaf} at four levels of I_{inc} (µmol m^{-2} s^{-1}) (b). C_c = 250 µmol mol^{-1}, leaf temperature = 25°C.

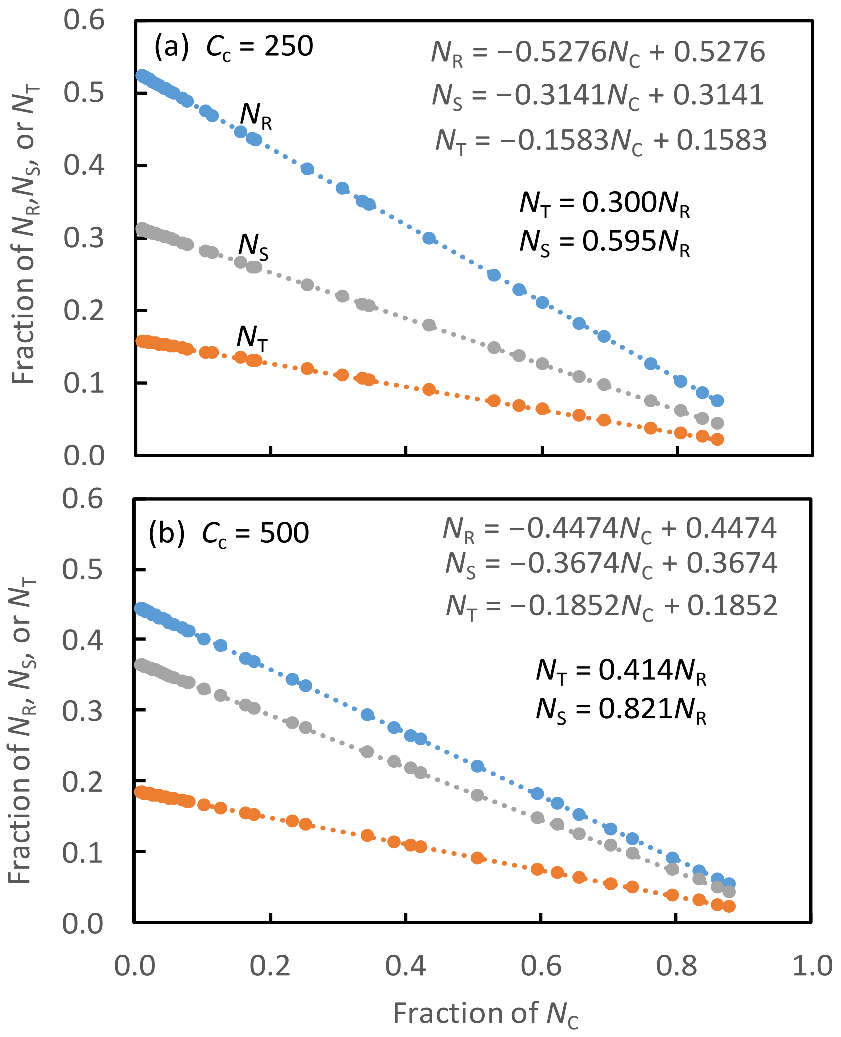

Fig. S2. Relationships between relative fractions of partitioning to N_C, N_R, N_T and N_S (leaf temperature = 25°C) under conditions of C_c = 250 μmol mol^{-1} (a) and C_c = 500 μmol mol^{-1} (b). Points are model predictions, and lines represent the fitted relations as given in the figure.

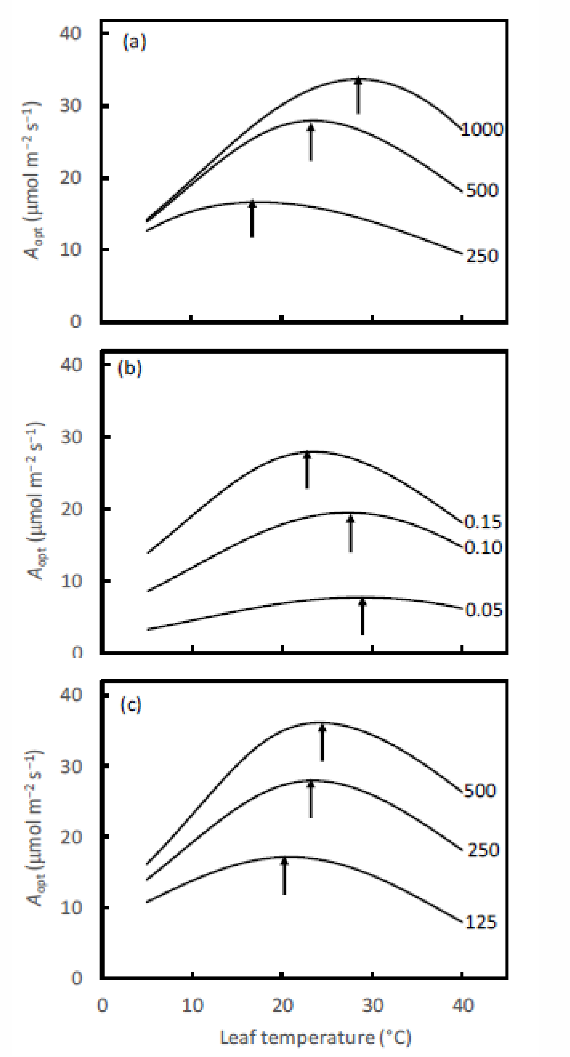

Fig. S3. Model-generated leaf photosynthesis rate under the optimum nitrogen partitioning, A_{opt}, in response to leaf temperature, at three levels of I_{inc} (µmol m^{-2} s^{-1}) when C_c = 250 µmol mol^{-1} and N_{leaf} = 0.15 mol m^{-2} (a), at three levels of N_{leaf} (mol m^{-2}) when C_c = 250 µmol mol^{-1} and I_{inc} = 500 µmol m^{-2} s^{-1} (b), and at three levels of C_c (µmol mol^{-1}) when N_{leaf} = 0.15 mol m^{-2} and I_{inc} = 500 µmol m^{-2} s^{-1} (c). Arrows indicate the optimum temperature, at which the predicted A_{opt} is maximal.

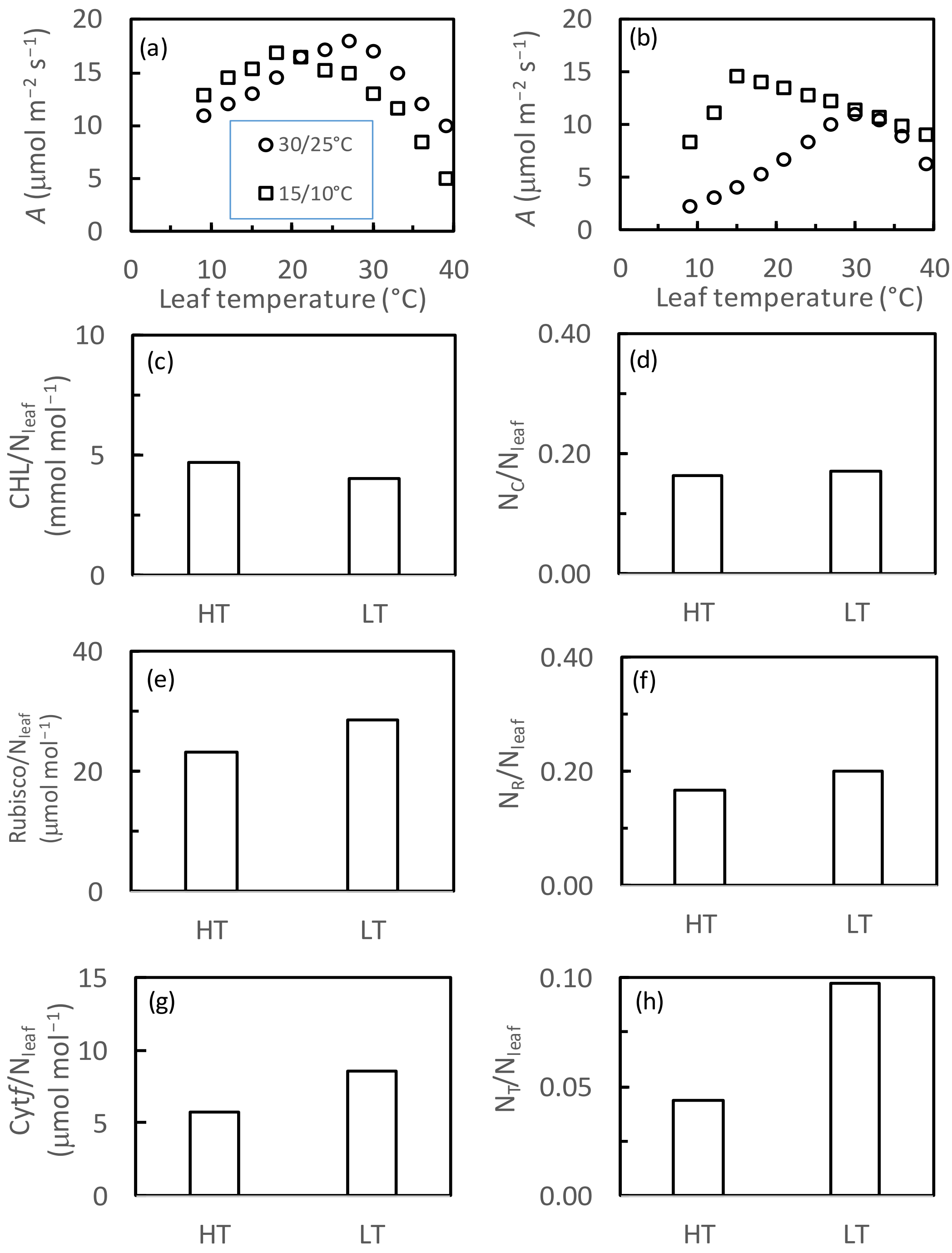

Fig. S4. Observed (a) and simulated (b) net CO_2 assimilation rate (A, at 1500 μmol m^{-2} s^{-1} irradiance and ambient CO_2 level) in response to measurement temperature, and observed amounts of photosynthetic proteins per unit N_{leaf} (c, e, g) vs predicted nitrogen in equivalent protein compounds per unit N_{leaf} (d, f, h) in leaves of spinach plants grown in low temperature (LT, day/night = 15/10°C), and high temperature (HT, 30/25°C). Other growth conditions: I_{inc} = 230 μmol m^{-2} s^{-1}, N_{leaf} = 0.1269 and 0.0811 mol m^{-2} for LT and HT, respectively (experimental data from Yamori et al. 2005).

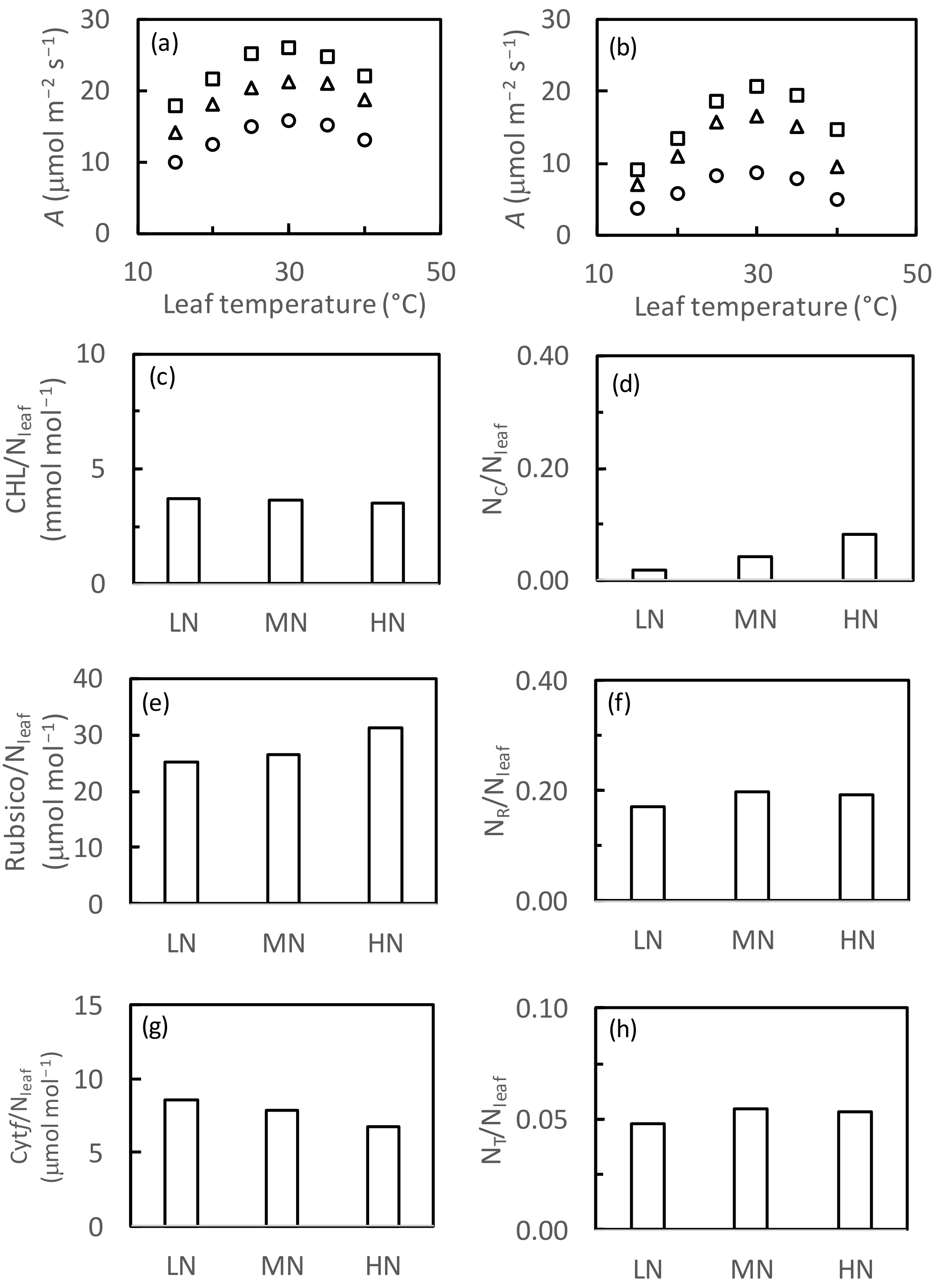

Fig. S5. Observed (a) and simulated (b) net CO_2 assimilation rate (A, at 1500 μmol m^{-2} s^{-1} irradiance and ambient CO_2 level) in response to measurement temperature, and observed amounts of photosynthetic proteins per unit N_{leaf} (c, e, g) vs predicted nitrogen in equivalent protein compounds per unit N_{leaf} (d, f, h) in leaves of four species grown in low nitrogen (LN), medium nitrogen (MN), and high nitrogen (HN). Other growth conditions: day/night temperature = 27/22°C, I_{inc} = 550 μmol m^{-2} s^{-1}0, N_{leaf} ranged 0.0419-0.0913, 0.071-0.142, and 0.1103-0.1684 mol m^{-2} for LN, MN, and HN levels, respectively (experimental data from Yamori et al. 2011 with more details therein). All data represented in this figure are the average of the four species. In panels (a) and (b), symbols "circles", "triangles" and "squares" represent LN, MN and HN leaves, respectively.

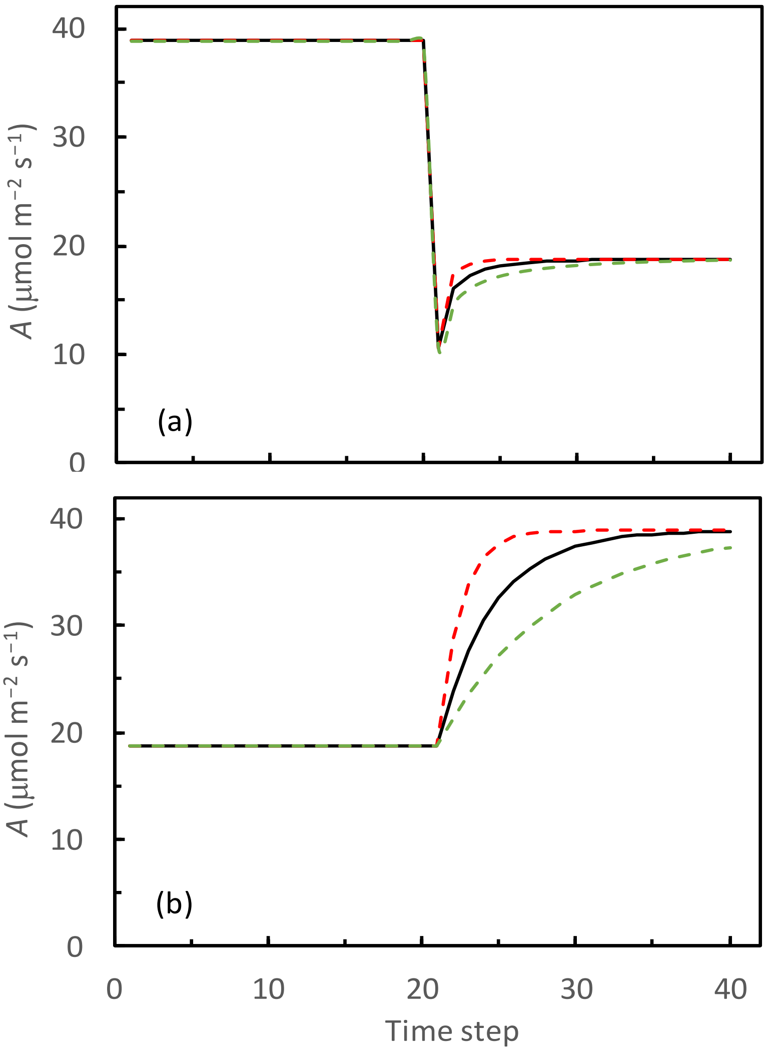

Fig. S6. Kinetics of net leaf photosynthesis A when incoming irradiance (I_{inc}) is abruptly (a) decreased from 1000 to 300 µmol m^{-2} s^{-1} or (b) increased from 300 to 1000 µmol m^{-2} s^{-1} at the 20th time step of simulation, simulated using eqn (9) with constant τ being 2 (orange dashed curve), 4 (black solid curve) or 8 times (green dashed curves) the time-step length, respectively. In this simulation, T_{leaf} = 25°C and N_{photo} = 0.1 mol m^{-2}. For the simulation shown in each panel, the optimum partitioning of N_{photo} for the given irradiance condition was used as the initial state.

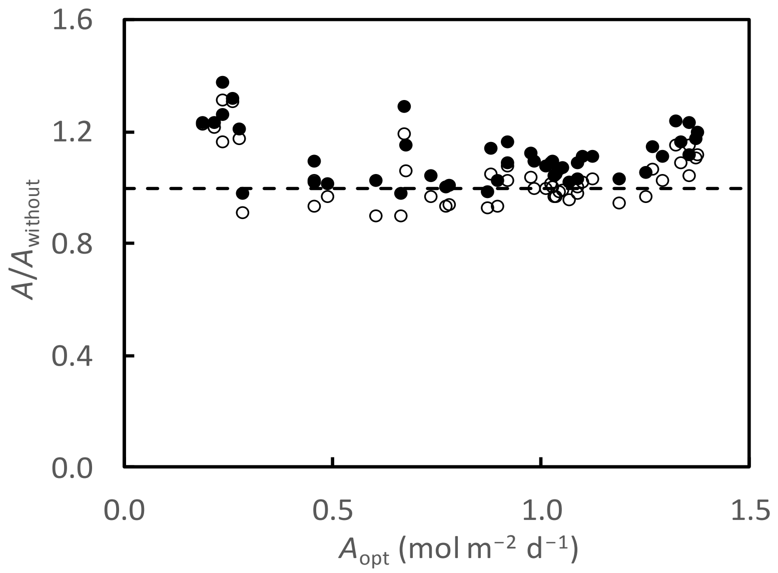

Fig. S7. The ratio of daily photosynthetic rate simulated assuming acclimation (A) to daily rate simulated without acclimation assumed ($A_{without}$), plotted against daily photosynthetic rate with the instantaneous optimum N partitioning (A_{opt}) over a period of ca 50 days. The variation of A_{opt} reflects the favourableness of daily weather (especially irradiance) for photosynthesis. The figure shows the result of upper leaves of a canopy, using two values of time constant τ, 40 min (filled symbols) and 80 min (open symbols), to simulate A. The results for middle and bottom leaves had a similar pattern, and therefore, are not shown in the figure.
